# Canadian Colorectal Cancer Screening Guidelines: Do They Need an Update Given Changing Incidence and Global Practice Patterns?

**DOI:** 10.3390/curroncol28030147

**Published:** 2021-04-21

**Authors:** Anastasia Kalyta, Mary A. De Vera, Stuart Peacock, Jennifer J. Telford, Carl J. Brown, Fergal Donnellan, Sharlene Gill, Jonathan M. Loree

**Affiliations:** 1 Division of Medical Oncology, BC Cancer/University of British Columbia, Vancouver, BC V5Z 4E6, Canada; akalyta@student.ubc.ca (A.K.); sgill@bccancer.bc.ca (S.G.); 2 Faculty of Pharmaceutical Sciences, University of British Columbia, Vancouver, BC V6T 1Z3, Canada; mdevera@mail.ubc.ca; 3 Cancer Control Research, BC Cancer, Vancouver, BC V5Z 4E6, Canada; speacock@bccrc.ca; 4 Division of Gastroenterology, University of British Columbia, Vancouver, BC V5Z 1M9, Canada; jtelford@telus.net (J.J.T.); fergal.donnellan@vch.ca (F.D.); 5 Division of General Surgery, St. Paul’s Hospital, Vancouver, BC V6Z 1Y6, Canada; cbrown@providencehealth.bc.ca

**Keywords:** colon, rectal, screening, early-onset, neoplasm, public healthcare

## Abstract

Colorectal cancer (CRC) is the third most commonly diagnosed cancer and second leading cause of cancer death in Canada. Organized screening programs targeting Canadians aged 50 to 74 at average risk of developing the disease have contributed to decreased rates of CRC, improved patient outcomes and reduced healthcare costs. However, data shows that recent incidence reductions are unique to the screening-age population, while rates in people under-50 are on the rise. Similar incidence patterns in the United States prompted the American Cancer Society and U.S. Preventive Services Task Force to recommend screening begin at age 45 rather than 50. We conducted a review of screening practices in Canada, framing them in the context of similar global health systems as well as the evidence supporting the recent U.S. recommendations. Epidemiologic changes in Canada suggest earlier screening initiation in average-risk individuals may be reasonable, but the balance of costs to benefits remains unclear.

## 1. Introduction

Colorectal cancer (CRC) is the third most commonly diagnosed cancer and second leading cause of cancer death both in Canada and worldwide [1,2]. It is estimated to account for 26,900 new diagnoses and 9700 deaths among Canadians in 2020 [3]. In Canada and the U.S., those over age 50 are most affected, but decreasing incidence in the past several decades has been attributed to increased participation in screening [2,4,5,6,7,8,9]. In line with published recommendations, organized screening programs targeting average-risk individuals aged 50 to 74 occur in every Canadian province and the Yukon territory [2]. While about 75% of Canadian CRC cases are diagnosed in Stages I–III, increased uptake of screening can further increase chances of diagnosis at a curable stage and improve survival [10].

Unsurprisingly, benefits are isolated to the age groups targeted by screening programs. While overall rates have decreased, significant increases in CRC incidence have been reported in individuals under 50 in both Canada [11,12] and the U.S. [6,7,13,14]. The practice of screening from age 50 has been upheld in iterations of Canadian and American guidelines until a 2018 American Cancer Society (ACS) update recommending screening begin at age 45 [8]. This was again endorsed in 2020 in a draft guideline statement by the U.S. Preventive Services Task Force [15]. We undertook a review of Canadian screening practices, placing them in the context of global patterns of screening and cancer incidence to help frame whether lowering the screening age in Canada may be a reasonable consideration.

## 2. Background

### 2.1. Colorectal Cancer in Canada

The lifetime probability of developing CRC in Canada is 7% for males and 5.6% for females [2]. Overall CRC incidence and mortality have been decreasing in Canada in recent years [2,12,16] but age-stratified data shows increases in the under-50 age group. Projections for 2019 show that while 56% of new diagnoses were in individuals aged 50–74, 7% of new cases and 4% of CRC-related deaths occurred in those under 50 [2].

### 2.2. Role of Screening

The basis of CRC screening is that most colorectal cancers stem from benign adenomatous polyps [17]. Screening reduces CRC incidence and mortality by identifying and treating adenomatous polyps and early-stage tumors before symptoms begin. It has been shown to improve patient outcomes [18] while remaining cost-effective compared to not screening [19,20]. Organized screening programs are defined as those with a systematic approach to reaching participants and a specific screening methodology, including tests used, timelines, target age group and follow-up on positive tests [21]. These organized regional, population-based programs are more effective than opportunistic screening, which occurs when an individual requests their own screening test or is recommended to screen by a healthcare provider [18,22]. Organized CRC screening programs have been developed across Canada since 2007 and every region either has or is currently planning an established program [2,23].

### 2.3. Common Screening Tests

Currently recommended screening tests involve either a structural examination of the colon or detection of bleeding from lesions. Occult blood is detected using non-invasive stool-based tests, primarily the fecal immunochemical test (FIT) or guaiac fecal occult blood test (gFOBT). The gFOBT identifies occult blood through peroxidase activity of heme while FIT uses antibodies to directly identify human globin in the stool [21,24]. While both are currently used in Canada, most provincial programs favor FIT. The gFOBT can yield false positives in individuals who recently consumed red meat, fruits or vegetables containing peroxidases or vitamin C [24,25]. FIT is not influenced by dietary factors [8] and has been shown to yield up to 13% higher participation rates than gFOBT [24,26,27,28]. This may be because FIT collection kits are easier to use, involve less handling of one’s stool, and require no diet or medication changes prior to screening [24]. FIT has higher sensitivity than gFOBT but is more expensive per test, and neither test poses direct risk to the user [8,21]. Analysis done for Ontario’s ColonCancerCheck program estimated the cost of FIT at $31.11 per test compared to $28.23 for gFOBT, but modeling ultimately found FIT to be more cost-saving when used in organized screening programs [29]. This is because FIT outperforms gFOBT in detecting large, pre-cancerous adenomas, thus preventing more eventual CRC cases and reducing treatment costs [28,29]. Structural examination options include flexible sigmoidoscopy and colonoscopy. While these are more invasive, require bowel preparation and have a risk of uncommon but serious complications, they offer the advantage of a longer time interval between tests [8]. A 2016 meta-analysis reports complication rates following screening colonoscopy to be 0.84 minor bleeds, 1.08 major bleeds (requiring hospitalization), 0.53 perforations and 0.02 deaths per 1000 patients [30].

## 3. CRC Screening in Canada

### 3.1. Guidelines Published in Canada

The Canadian Association of Gastroenterology (CAG) updated its screening guidelines for CRC in 2010, highlighting the need for regionally or provincially organized programs (Table 1). The CAG recommends screening with FIT (preferable) or high sensitivity gFOBT every two years, or sigmoidoscopy every ten years in individuals aged 50 to 75 with average risk of developing CRC. Screening in those aged 76 to 85 should be approached case-by-case, considering life expectancy and screening history, and is not recommended beyond age 85 [18]. The CAG suggests training non-physicians to perform sigmoidoscopy to keep up with demand [18], which is currently being done in Ontario [31].

The most recent primary care guidelines were published by the Canadian Task Force on Preventive Health Care in 2016 [9] (Table 1). They suggest screening for CRC from age 50 to 74 only, with cessation at age 75. The task force divides their recommendation to screen into grades, designating it as strong for individuals aged 60 to 74 and weak for 50 to 59. The weak recommendation is based on the rationale that lower incidence in the younger age group results in a lower absolute screening benefit [9]. In practice, the task force suggests health providers encourage all patients aged 60 to 74 to screen but discuss risks and benefits before offering screening 50 to 59-year-olds. Decision-aids have been published to help with this process.

Currently, little randomized controlled trial (RCT) data is available on the efficacy of FIT over gFOBT. A systematic review carried out by Cancer Care Ontario [24] suggests FIT is superior in terms of patient uptake and sensitivity for CRC and advanced adenomas, while having similar specificity and positive predictive value as gFOBT [24]. As FIT’s performance depends on the cut-off value used [24], the task force recommends screening programs using FIT provide guidelines for individual laboratories to set their own cut-off values [9]. Programs electing to use gFOBT should use only high-sensitivity gFOBT [9]. 

Neither guideline recommends colonoscopy as a screening tool, citing a need for RCT evidence showing clear superiority over other test options [30]. The high resource cost, increased risk and long wait lists for colonoscopy in Canada are also important considerations. Rather, it is used as a follow-up test for any positive screen. In the case of a negative follow-up colonoscopy, screening in average-risk individuals should resume in 10 years or earlier if symptoms appear [18].

### 3.2. Provincially Organized Screening Programs

Screening programs in Canada are delivered provincially and are all similar (Table 2). Each province screens individuals aged 50 to 74 and most use FIT every two years as the main modality. Exceptions include Alberta, where FIT is done annually and Manitoba, which uses gFOBT. Ontario also offers sigmoidoscopy every 10 years for screening by registered nurses trained to perform these tests [31]. To participate, most provinces require individuals to be referred by a clinician or request enrolment themselves. Saskatchewan, New Brunswick and Nova Scotia automatically enroll participants when they turn 50 and mail out invitations or testing kits every two years [33]. 

### 3.3. Screening Participation in Canada

**Figure 1:**
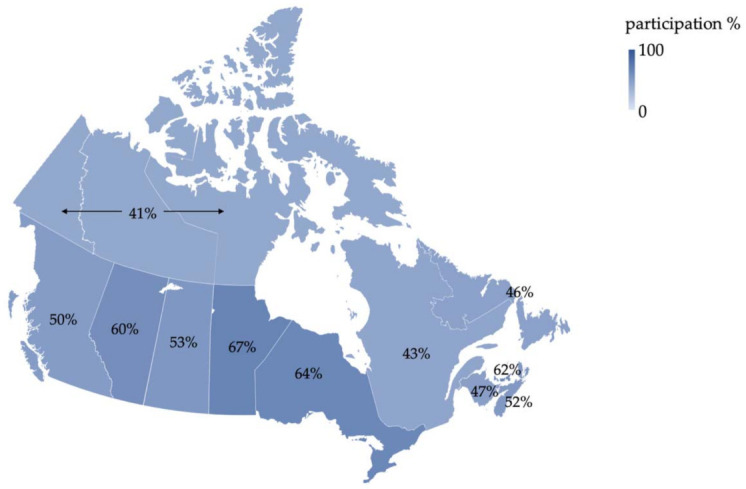
Proportion of eligible Canadians up to date with screening (defined as having had a fecal test in the last two years or colonoscopy/sigmoidoscopy in the last ten), based on self-reported data from the 2012 Canadian Community Health Survey [34].

Self-reported data from 2012 shows 55.2% of Canadians aged 50–74 were up to date with CRC screening, having had a fecal test in the last two years or colonoscopy/sigmoidoscopy in the last ten [34]. Provincial participation rates ranged between 41.3% in the territories and 67.2% in Manitoba, the first province to implement population-based testing [34] (Figure 1). Some provincial programs had been implemented more recently than others at data collection, likely contributing to this variation. Younger age, lower income and education level, living in a rural area, smoking and self-identifying as an immigrant are further predictors for decreased screening participation [34,35]. A study using the Manitoba Cancer Registry showed increased CRC mortality among residents of lower-income areas, highlighting a need to focus on screening access and promotion in lower-income populations [34,36].

## 4. CRC Screening in the United States

In contrast to Canada, CRC screening in the U.S. is mainly opportunistic with some regionally-organized programs [21]. In 2015, 62.4% of Americans were up to date with screening, but this number drops to 25.1% in the uninsured population [37]. As in Canada, guidelines are published by various organizations to guide health providers, the most recent of which is a 2018 update from the ACS (Table 1). The major revision from the ACS is a recommendation to begin screening at age 45, rather than 50 as in previous U.S. guidelines [32,38,39]. This is based on multiple factors, including screening test efficacy, increasing burden of CRC in Americans under 50 and modeling studies predicting reduced mortality with screening at 45 [8]. Financial implications were not considered. The six screening strategies recommended are: annual FIT or high-sensitivity gFOBT, multitarget stool DNA test every 3 years, sigmoidoscopy every 5 years, colonoscopy every 10 years or CT colonography every 5 years. The ACS cites increased screening adherence among patients who are given options, and thus recommends letting patients choose rather than endorsing a specific test [8]. Screening is recommended until age 75, with individuals aged 76 to 85 approached case-by-case.

In October 2020, the U.S. Preventive Services Task Force (USPSTF) released a draft recommendation statement also advocating for screening to begin at age 45 in average-risk adults [15] (Table 1). Recommended screening modalities are in line with previous USPSTF guidelines [32]. The new position taken by the USPSTF is that beginning screening at age 45 offers moderate net benefit in the form of life-years gained and reduced CRC mortality balanced with low harms from screening [15].

## 5. CRC Screening in Europe

The Council of the European Union published a recommendation for population-based CRC screening in 2003, suggesting fecal occult blood testing in individuals aged 50 to 74 [40]. In 2010, the European Commission expanded this with an extensive set of quality-control guidelines for screening [41]. They do not endorse specific testing methods, but outline FIT, sigmoidoscopy and colonoscopy as reasonable options for average-risk individuals [41,42]. The European Society for Medical Oncology (ESMO) published a clinical practice guideline in 2013 based on the European Commission’s work [43]. ESMO’s guidelines recommend screening with gFOBT in individuals aged 50 to 74 at least every 2 years [43,44]. FIT at least every 3 years or sigmoidoscopy every 10–20 years (most appropriately done in individuals aged 55 to 64) are also options while colonoscopy is not recommended for screening [43].

Despite the presence of Europe-wide guidelines, screening practices differ widely among nations. Not every country has adopted a systematic screening program and those that have differ in tests offered, screening age and frequency [21,45]. We selected a few health systems comparable to Canada to compare and contrast (Table 3).

### 5.1. France

France launched a national CRC screening program in 2008, originally offering gFOBT but switching to FIT in April 2015 [49]. Individuals aged 50 to 74 at average risk of developing CRC undergo screening every 2 years and a positive screen is followed up by colonoscopy [50]. Patients are recruited via mailed letters inviting them to obtain an at-home kit from their family doctor [50]. In the first two years after its launch, regional uptake of the program covered 57% of the target population in France and 34.3% of invited individuals partook in screening [50].

### 5.2. The Netherlands

Population-based screening was launched in the Netherlands in 2014, with staggered inclusion of different age groups over 5 years. Individuals turning 63, 65, 67, 75 and 76 were recruited in the first year, with 71.3% participating [51]. Program performance in the first year was monitored in real-time, allowing organizers to identify higher than expected participation and false-positive rates in the early months. The positive result cut-off value was subsequently increased, allowing positive predictive value to be optimized and colonoscopy demand reduced to a manageable level [51]. Currently, the program automatically mails a FIT kit every two years to individuals aged 55 to 75 [51,52].

### 5.3. United Kingdom

The U.K.’s National Health Service began the Bowel Cancer Screening Program in 2006, originally mailing biennial at-home gFOBT kits to individuals aged 60 to 69 and expanding to ages 60 to 74 in 2010 [47]. A 2018 decision to begin screening at 50 has not yet been implemented [53]. Scotland has slightly different guidelines and already screens from age 50 to 74 [47]. FIT replaced gFOBT as the main test method in 2018 (2020 in Northern Ireland [46]) and an additional, one-time sigmoidoscopy at age 55 is offered in England only [47,53]. English residents are sent sigmoidoscopy invitations automatically when they turn 55, while those aged 55 to 60 who have not participated can ask for a referral [47]. Participation rates for gFOBT screening in 2012–2015 ranged from 49.8% in Northern Ireland to 57.9% in England [48] and participation in the in the first 14 months of the sigmoidoscopy program was 43.1% [54]. Higher rates are expected after the switch to FIT, as suggested by a 2014 FIT pilot study in England [47]. The pilot showed increased overall participation in individuals randomly assigned to FIT over gFOBT and an almost doubled participation in FIT among individuals who previously did not screen at all [26].

## 6. Early-Onset CRC in the U.S.

Age-stratified data shows decreasing CRC incidence and mortality in Americans over 50 from 2000 to 2013 [5,6]. However, incidence and mortality before age 50 have increased by 22% and 13% respectively in this time frame, with an annual percent change (APC) in incidence of 1.6 [5]. According to the World Health Organization’s GLOBOCAN estimates for 2018, age-standardized incidence among Americans under 50 is 5.7 per 100,000 [1,55] (Table 4) and 2017 data shows men and women under 50 making up 11% and 10% of new CRC cases respectively [5]. 

**Figure 2:**
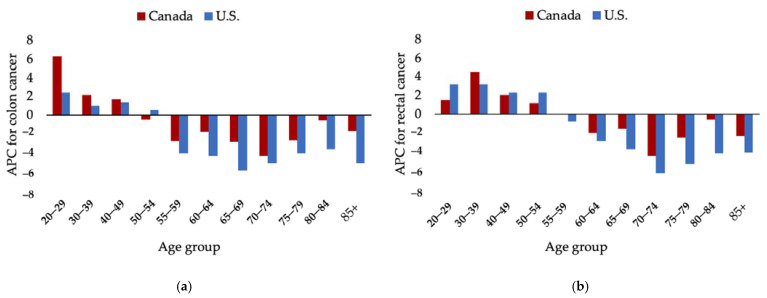
Comparison of annual percent change (APC) by age-group between Canada [56] and the U.S. [13] for colon (**a**) and rectal (**b**) cancers. APCs based on most recent published trends.

The ACS recommendation to lower the screening age to 45 is influenced by several studies highlighting increasing CRC rates in Americans under 50 and a related birth-cohort effect. Colon cancer incidence in Americans aged 40 to 49 has been increasing since 1996 with an APC of 1.3, while rectal cancer has been trending up since 1991 with an APC of 2.3 [13] (Figure 2). Cohort analysis shows Americans born around 1990 have a two times and four times higher risk for developing colon and rectal cancer respectively than those born around 1950 [8,13].

As noted by Abualkhair et al., reported CRC rates in individuals under 50 comprise mostly cases discovered due to symptomatic presentation or early screening of those at above-average risk [57]. Thus, these rates are an under-estimation as pre-symptomatic, early-stage cancers cannot be diagnosed until individuals become eligible for screening at age 50 [57]. A 46.1% incidence jump observed between Americans aged 49 and 50 suggests that many undiagnosed CRC cases may already exist in the under-50 population [57].

## 7. Early-Onset CRC in Canada

Similar to the U.S., Canada has seen decreased CRC incidence in adults over 50 from 2000 to 2015 [11] while rates in individuals under 50 are on the rise [11,56,58]. This may be partially explained by screening. Brenner et al. [56] analyzed CRC rates before and after a 2004 screening guideline publication from the CAG and Canadian Digestive Health Foundation [59], observing a significant decline in CRC rates in the over-50 age group after 2004, but no significant change in the under-50 group.

Colorectal cancer incidence in Canadians under 50 has been increasing with overall APCs of 3.5 for men and 4.5 for women according to most recent data [11]. GLOBOCAN estimates place the age-standardized incidence in Canadians under 50 at 5.4 per 100,000, not far off from the U.S. rate [1,55] (Table 4). The 40 to 49 age group is the main driver of rising early-onset CRC rates [11,56] and a 2017 study of Canadian cancer registry data shows similar trends to those cited in the ACS 2018 guideline update [56]. Colon cancer incidence in Canadians aged 40 to 49 has been increasing since 2003 with an APC of 1.66 and rectal cancer has been rising since 1996 with an APC of 2.05 [56] (Figure 2).

A birth-cohort effect is also seen in Canada: cohorts prior to 1980 have similar rates while individuals born after 1980 have more than double the risk of developing CRC [11]. As in Siegel et al.’s U.S. study [13], the Canadian authors cite obesity in later birth cohorts as a contributor to CRC rates, as both follow an upward trend [56]. Both the Canadian and American studies suggest younger screening ages should be considered but also call for action on rising obesity rates in young people [13,56].

## 8. Modeling Studies—U.S.

The 2018 ACS guideline update was largely based on predictions from the National Cancer Institute’s MISCAN-Colon model, updated with early-onset CRC incidence as described by Siegel et al. in 2017 [60,61]. The model predicts screening starting at 45 instead of 50 will result in more life-years gained for each of the six ACS-endorsed strategies, with manageable increases in colonoscopy demand [60]. Colonoscopy every 10 years from age 45 to 75 is the top option according to the new model, yielding 25 added life-years and a 17% increase in colonoscopy demand per 1000 people screened [60]. The model also found annual FIT, sigmoidoscopy every 5 years or CT colonography every 5 years from age 45 to 75 to be similarly effective, offering at least 90% of the life-years gained by colonoscopy [60]. The ACS did not assess economic implications, stating that cost and resource availability are not considered in their recommendation process and will vary regionally [8]. A 2019 modeling study predicts screening per the new guideline to be cost-effective, but less so than increasing screening uptake among individuals over 50 or those with above-average risk [62].

## 9. Modeling Studies—Canada

The Canadian Task Force on Preventive Health Care’s 2016 guidelines [9] include analysis of the economic impact of CRC screening. Two Canadian modeling studies based on individuals aged 50 to 75 suggest annual FIT or colonoscopy every 10 years to be most effective and the best choices economically [4,63,64]. Telford at al. found any screening strategy to be cost-effective compared to no screening, with colonoscopy having both the highest benefit and cost [63]. Heitman et al. endorsed annual FIT for having better efficacy and lower cost than almost all other available modalities [64]. Their model predicts a 71% reduction in CRC incidence and a 74% reduction in deaths over the lifetime of 100,000 Canadians, along with saving $68 CAD per individual when using FIT with median accuracy [64]. Even though a single instance of FIT is less effective than colonoscopy, authors state that yearly FIT offers more opportunities to identify abnormalities that may be missed in the 10-year interval between colonoscopies [64]. In simulations using the Canadian Partnership Against Cancer’s Cancer Risk Management Model (now called OncoSim), colonoscopy either at 10-year intervals or once per lifetime appears more effective and cheaper than FIT [4]. However, the task force points out that colonoscopy has not been confirmed as more effective by RCT data and cannot be widely used for screening without first increasing healthcare system capacity, which simulated costs do not account for [4].

A separate study using the Cancer Risk Management Model reports similar predictions for screening in ages 50 to 74 but also assesses stool-based screening beginning at age 45 [65]. The model predicts that biennial FIT or gFOBT in ages 45 to 74 yields 20 additional life-years per 1000 people screened along with a 10% increase in colonoscopy demand, compared to ages 50 to 74 [65]. Screening from age 45 would increase costs by 13% and 14% for FIT and gFOBT respectively [65]. These results are similar to the model used in the ACS guideline update, which predicted yearly FIT or gFOBT beginning at 45 to yield 26 additional life-years and a 12% and 14% increase in colonoscopy demand respectively per 1000 people screened [8,60]. The ACS states that U.S. colonoscopy resources are sufficient to handle this increase, but further research is needed to see how such a change would affect the Canadian healthcare system.

## 10. Future Screening Strategies

Commercial stool-based tests vary widely in performance and structural tests come with inherent risks and additional effort for patients and healthcare systems [8]. New screening strategies are being developed, many of which are moving away from relying on polyp detection.

One of the more established new screening methods is the mSEPT9 DNA test. This test detects methylated Septin9 DNA in the blood, a known biomarker shed by colorectal tumors. The test is approved by the U.S. FDA for screening only when an individual rejects all other guideline-recommended screening options [8,66].

Liquid biopsy techniques involve analysis of blood or other body fluids for various biomarkers indicative of cancer. These may be found in circulating tumor cells, tumor DNA, microRNA, long non-coding RNA or proteins [67]. Liquid biopsy-based tests have potential to facilitate screening and diagnosis as well as predict relapse or metastasis, monitor progression and treatment response, and identify chemotherapy-resistant cancers [67].

GRAIL is a recent initiative developing a liquid biopsy test to screen for multiple cancers at once. This involves sequencing circulating tumor DNA from blood and referencing it to a database of abnormal methylation patterns associated with various cancers [68]. The company is currently running their Circulating Cell-Free Genome Atlas study to identify these patterns in early-stage cancers [69].

Another possibility for screening is use of mass spectrometry to identify metabolites associated with CRC in blood. One study has identified sex-specific metabolite profiles for CRC, which vary between disease stage, and between individuals with recurrent Stage II CRC and those without [70]. Authors report a positive predictive value for CRC of over 89% and ability to detect very early-stage cancer with these particular profiles. However, they point out that their profiles differ from previous CRC metabolome studies [70].

## 11. Conclusions

Colorectal cancer screening delivered through provincially organized programs contributes to decreasing CRC incidence and mortality among Canadians of screening age and continued promotion of these programs will further their benefit. As Canadian published screening guidelines remain similar and align with those of comparable health systems, it may be logical for organizations to endorse guidelines from other nations rather than publishing new ones, and focus efforts on improving access to and participation in existing screening programs.

While CRC rates in Canadians and Americans over 50 have decreased since screening implementation, early-onset disease is on the rise. Similarities in the burden of early-onset CRC, particularly in the 40 to 49 age group, in Canada and the U.S. suggests that beginning screening at 45 as outlined by the ACS and USPSTF may be something to consider in Canada. However, more robust modeling is needed to assess the costs and benefits of such a change, keeping in mind Canada’s publicly funded healthcare system and long colonoscopy wait times.

## Figures and Tables

**Table 1:** Most recent published guidelines for colorectal cancer screening in average-risk individuals in Canada and the United States.

Publishing Organization	Last Updated	Ages Targeted	Recommended Screening Test Options	Recommended Follow-Up for Positive Screen
Canadian Task Force on Preventive Health [9]	2016	60–74 (strong recommendation; screening should be offered to all), 50–59 (weak recommendation; screening to be offered after discussing harms and benefits), no screening at 75+	FIT or HS-gFOBT q2 yrs, FS q10 yrs	Colonoscopy
Canadian Association of Gastroenterology [18]	2010	50–75, 76–85 case-by-case	FIT (preferred) or HS-gFOBT q1–2 yrs, FS q10 yrs	Colonoscopy
U.S. Preventive Services Task Force (draft posted October 2020) [15]	2020	45–75, 76–85 case-by-case	FIT or HS-gFOBT q1 yr, DNA-FIT q1–3 yrs, FIT q1 yr, plus FS q10 yrs, FS q5 yrs, colonoscopy q10 yrs, CTC q5 yrs	Colonoscopy
American Cancer Society [8]	2018	45–75 (qualified recommendation), 50–75 (strong recommendation), 76–85 case-by-case	FIT or HS-gFOBT q1 yr, DNA-FIT q3 yrs, FS q5 yrs colonoscopy q10 yrs, CTC q5 yrs,	Colonoscopy
U.S. Preventive Services Task Force [32]	2016	50–75, 76–85 case-by-case	FIT or gFOBT q1 yr, FIT-DNA q1–3 yrs, FIT q1 yr plus FS q10 yrs, FS q5 yrs, colonoscopy q10 yrs, CTC q5 yrs	Colonoscopy

FIT = fecal immunochemical test, HS-gFOBT = high-sensitivity guaiac fecal occult blood test, DNA-FIT = multitarget stool DNA test, FS = flexible sigmoidoscopy, CTC = computed tomography colonography.

**Table 2:** Summary of provincially-organized screening programs for individuals at average risk of developing colorectal cancer.

Province	Program Name [23,33]	Organization Responsible	Program Start	Ages Targeted	Screening Tests Offered	Enrolment in Program	Cut-off for Positive FIT (ng/mL) [23]
Alberta	Alberta Colorectal Cancer Screening Program	Alberta Health Services	2009	50–74	FIT q1 yr	Referral by clinician	75
British Columbia	Colon Screening Program	BC Cancer	2013	50–74	FIT q2 yrs	Referral by clinician, Northern Health Authority does not participate	50
Manitoba	ColonCheck	CancerCare Manitoba	2007	50–74	gFOBT q2 yrs (requires 3 different samples)	Referral by clinician or individual requests online/by phone	N/A
New Brunswick	New Brunswick Colon Cancer Screening Program	New Brunswick Cancer Network	2014	50–74	FIT q2 yrs	Invitations mailed q2 yrs once individual turns 50	100
Newfoundland and Labrador	Newfoundland and Labrador Colon Cancer Screening Program	Eastern Health	2012	50–74	FIT q2 yrs	Referral by clinician or individual requests online/by phone	100
Northwest Territories	Organized screening program in planning stages	Northwest Territories Health and Social Services Authority	N/A	50–74	FIT q1–2 yrs	Referral by clinician	75
Nova Scotia	Colon Cancer Prevention Program	Nova Scotia Health Authority	2009	50–74	FIT q2 yrs	Kits mailed q2 yrs once individuals turn 50	100
Ontario	ColonCancerCheck	Cancer Care Ontario	2008	50–74	FIT q2 yrs or FS q10 yrs (can be done by registered nurse)	Referral by clinician or individual requests by phone	Not reported
Prince Edward Island (PEI)	Colorectal Cancer Screening Program	Health PEI	2011	50–74	FIT q2 yrs (requires 2 different samples)	Individual requests online/by phone	100
Quebec	Colorectal Cancer Screening Program (still in pilot phase)	Ministère de la Santé et des Services Sociaux	Pilot started in 2011	50–74	FIT q2 yrs	Referral by clinician	175
Saskatchewan	Screening Program for Colorectal Cancer	Saskatchewan Cancer Agency	2009	50–74	FIT q2 yrs	Kits mailed q2 yrs once individuals turn 50	100
Yukon	ColonCheck Yukon	Government of Yukon Health and Social Services	2017	50–74	FIT q2 yrs	Referral by clinician or individual requests by phone	100
Nunavut	Organized program implementation is in-progress	Nunavut Department of Health	2018	50–74	FIT q2 yrs	Opportunistic screening available	Not reported

FIT = fecal immunochemical test, gFOBT = guaiac fecal occult blood test.

**Table 3:** Average-risk colorectal cancer screening guidelines for select European nations.

Country	Organization Responsible	Program Start/Updates	Ages Targeted	Screening Tests Offered	Follow-Up Test for Positive Screen	Participant Recruitment
United Kingdom [46,47,48]	National Health Service	Started in 2006, updated in 2018	60–74 in England, Wales and Northern Ireland, 50–74 in Scotland. A 2018 decision to start at 50 U.K.-wide has not yet been implemented.	FIT q2 yrs (gFOBT in Northern Ireland), Bowelscope program offers one-time FS at age 55 in England only	Colonoscopy	Kits mailed to eligible individuals
France [49,50]	French Ministry of Health and National Cancer Institute	2008, switched to FIT in 2015	50–74	FIT q2 yrs	Colonoscopy	Invitations mailed to eligible individuals; kits obtained from family doctor
Netherlands [51,52]	National Institute for Public Health and the Environment	2014	55–75	FIT q2 yrs	Colonoscopy	Kits mailed to eligible individuals

FIT = fecal immunochemical test, gFOBT = guaiac fecal occult blood test, FS = flexible sigmoidoscopy.

**Table 4:** Comparison of incidence and mortality data in Canada and the U.S.

Country	Age Group	Incidence (ASIR per 100,000) [1,55]	Mortality (ASMR per 100,000) [1,55]
Canada	All ages	31.5	10.1
	Over 50	135.6	47.0
	Under 50	5.4	0.91
U.S.	All ages	25.6	8.2
	Over 50	105.0	35.0
	Under 50	5.7	1.5

ASIR = estimated age-standardized incidence rate for 2018, standardized to a world standard population. ASMR = estimated age-standardized mortality rate for 2018, standardized to a world standard population.

## Data Availability

No new data were created or analyzed in this study. Data sharing is not applicable to this article.
